# Geological Interpretation of PSInSAR Data at Regional Scale

**DOI:** 10.3390/s8117469

**Published:** 2008-11-24

**Authors:** Claudia Meisina, Francesco Zucca, Davide Notti, Alessio Colombo, Anselmo Cucchi, Giuliano Savio, Chiara Giannico, Marco Bianchi

**Affiliations:** 1 Department of Earth Sciences - University of Pavia, Via Ferrata n 1, 27100 Pavia, Italy; E-Mails: francesco.zucca@unipv.it; davide.notti@dst.unipv.it; 2 ARPA Piemonte - Centro Regionale per le Ricerche Territoriali e Geologiche, via Pio VII, 9, 10135, Torino, Italy; E-Mails: a.colombo@arpa.piemonte.it; a.cucchi@arpa.piemonte.it; 3 Tele-Rilevamento Europa - T.R.E. S.r.l, Via Vittorio Colonna, 7, 20149 Milano, Italy; E-Mails: giuliano.savio@treuropa.com; chiara.giannico@treuropa.com; marco.bianchi@treuropa.com

**Keywords:** SAR interferometry, permanent scatterer, landslide, subsidence, Piemonte

## Abstract

Results of a PSInSAR™ project carried out by the Regional Agency for Environmental Protection (ARPA) in Piemonte Region (Northern Italy) are presented and discussed. A methodology is proposed for the interpretation of the PSInSAR™ data at the regional scale, easy to use by the public administrations and by civil protection authorities. Potential and limitations of the PSInSAR™ technique for ground movement detection on a regional scale and monitoring are then estimated in relationship with different geological processes and various geological environments.

## 1. Introduction

Synthetic Aperture Radar (SAR) images provide a complementary method to investigate ground deformation phenomena. In particular, differential SAR interferometry (DInSAR), based on the comparison of SAR images acquired at different times with slightly different look angles, can give a synoptic view of the deformation events projected along the sensor-target line of sight (LOS) on areas of hundreds to thousands of square kilometers [1]. Its intrinsic limits due to temporal and geometric decorrelations can be partly overcome by the use of the Permanent Scatterers interferometry technique (PSInSAR™), which relies on an advanced algorithm (developed by Politecnico of Milano) for the processing of data acquired by SAR sensors [2].

PSInSAR™ is an operational tool for precise ground deformation mapping on a sparse grid of phase stable radar targets (the so-called Permanent Scatterers, PS), acting as a “natural” geodetic network. This technique identifies, estimates and removes atmospheric distortions, leaving the PS displacement as the only contribution to the signal phase shift. The application of SAR data and, in particular, PSInSAR™ to detect and monitor surface deformations has advanced rapidly during the last decade, and it is now routinely applied to a wide range of natural hazards: landslides (detection and monitoring of landslides [3, 4], determination of landslide activity [5], modelling large slope instability [6]), subsidence (seasonal and long-term aquifer-system response to groundwater pumping [7], land surface deformation corresponding to seasonal groundwater fluctuation [8], natural subsidence and subsidence induced by urbanization [9, 10], quantification of the subsidence rate [11], estimation of non linear subsidence rate [12]) and tectonic motions [13]. Such an approach provides fast and updatable data acquisition over large areas, and can be integrated with conventional investigation methods (e.g. field surveys, airphotos interpretation). Of particular interest is the possibility to combine deformation measurements with geological data in a geographical information system (G.I.S).

This paper presents an application of PSInSAR™ technique for detecting and monitoring ground displacements at regional scale in the Piemonte Region. The aim of the project, supported by ARPA Piemonte and the National Civil Protection Department, is to apply and test the PSInSAR™ technique in order to: develop a methodological approach for the geological interpretation of the data at regional scale, verify the potentials and limitations of the technique for the detection of ground movements in relation to the different natural/anthropogenic processes and to the different geological environments, identify areas with ground deformations, where local authorities may concentrate future detailed geological studies and risk mitigation actions, and monitor large areas at relatively low costs.

## 2. Geological setting of the study area

**Figure 1.**
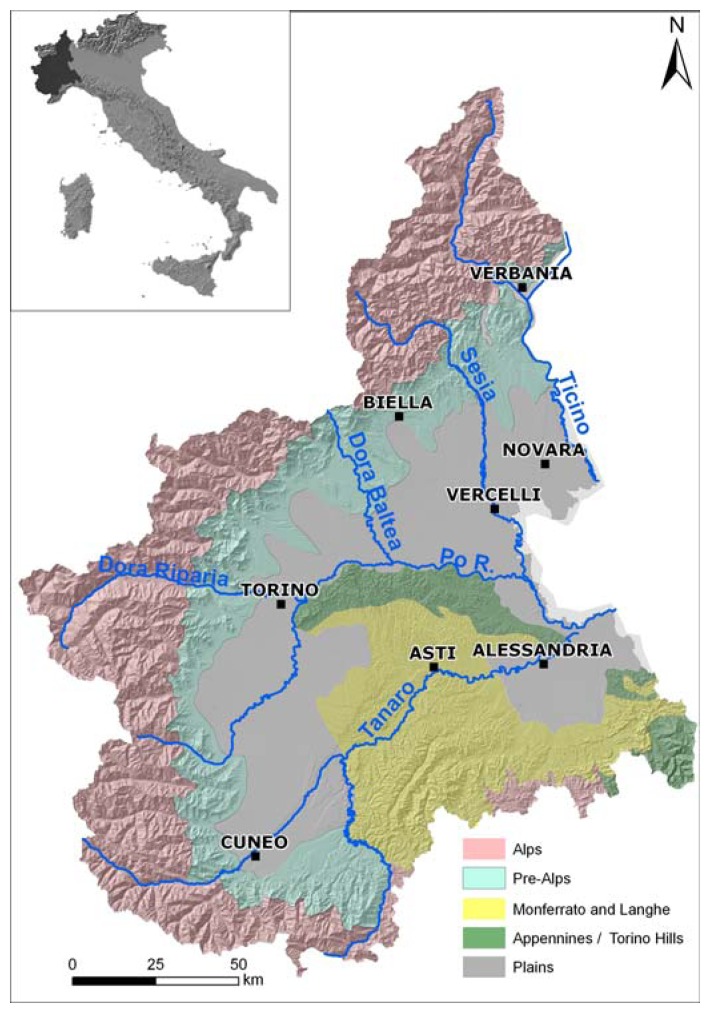
Study area.

The Piemonte Region, located in northwest Italy, has an extension of 25,000 km^2^ and it is significant with respect to different geological contexts (Alps, Apennines, Langhe and Monferrato and Plain, Figure 1).

The Alps are characterized by high slope gradients and the presence of foliated and massive rocks. The pre-alpine environment has more gentle and vegetated slopes (generally less than 30°) and widely distributed soil cover. The Langhe region is characterized by asymmetrical valleys, with steep southeast-facing slopes and gentle northwest-facing slopes and a monocline succession of marl and sandstone layers. The hilly southeastern part of Piemonte (Apennines and Torino hill) is characterized by sedimentary clay rock. Slopes in the argillaceous and marly successions are mantled with 1 to 6 m thick clayey-silty colluvial deposits.

The plain sector belongs to the Po River Plain and shows two morphological units: the upper plain, developed at the feet of the Alps, with rough permeable sediments (mostly gravels); the lower plain with finer sediments (sand, silt and clay) [14]. The upper and lower plains are separated by the Fontanile Line (spring subsurface water emergence produced by a sediment permeability decrease). Geophysical investigations show that the tectonic structure of the Apennines continued buried under the plain. Large synclines alternate with narrower anticlines, which give rise to isolated ridges (Torino and Monferrato hills). The Apennine margin is dominated by piedmont fans. The pediplan along the Alps presents large alluvial fans, often incised by rivers (e.g. Stura di Lanzo alluvial fan). Complex structures of glacial till are still clearly visible (e.g. the Ivrea morainic amphitheater).

The IFFI project (national landslide inventory) identified more than 34,000 slope instabilities [15], of various typologies related to the different geological settings. The alpine environment is characterized by the presence of rock-falls/topples, large complex landslides and deep seated gravitational deformations. In the Langhe area translational rock-block slides and shallow landslides make up the majority of landslides. The hilly southeastern part of Piemonte (Apennines/Torino hill) is affected by slow flows and complex landslides.

About 300 landslides are monitored (landslide regional monitoring network of ARPA Piemonte RERCOMF). The monitoring network includes many sites with few conventional instruments (inclinometers, piezometers, extensometers and topographic benchmarks). Many of them were installed in the Langhe after the November 1994 strong rainfall event. At a limited number of sites, where major landslides threaten large built-up areas or important structures, monitoring systems are more complex and include several types of instruments, with automated data recording and transmission, generally installed after 2000.

## 3. PSInSAR™ data

Conventional, differential, satellite repeat-pass InSAR is a methodology in which two radar scenes acquired over the same area at different times provide radar phase information that allows detection and measurement of sub-centimeter-scale ground movement in the form of a phase-change interferogram. The successful application of conventional InSAR to ground deformation studies is typically dependent upon a number of variables: availability of archival radar data to bracket the timing of the deformation event; suitable satellite baseline geometry; retrieval of coherent phase data, and identification and removal of phase changes unrelated to ground deformation, such as topography, residual satellite orbital errors, and atmospheric artifacts. Measurement of the radar phase change is made on a pixel-resolution (∼80 m^2^) basis with a full-cycle of phase change equivalent to one-half of the radar wavelength (5.6 cm for C-band radar), or 2.8 cm of radar line-of-sight (LOS) displacement.

In contrast, the permanent scatterer (PS) methodology utilizes the identification and exploitation of individual radar reflectors, or permanent scatterers, which are smaller than the resolution pixel cell and that remain coherent over long time intervals in order to develop displacement time series [2]. The resolution that is achieved by the identification of these PS targets effectively results in the creation of a data set consisting of many tiny “benchmarks”. A detailed description of the PS technique can be found in Ferretti *et al.* [2].

The advantages of the PS methodology are several: 1) good phase coherence is obtained from nearly all radar scenes regardless of geometrical baseline (perpendicular separation of the satellite positions), and long baseline interferometry with up to 1.6 km separation; 2) all available radar scenes in the archive can be exploited; and 3) atmospheric phase contributions can be estimated and removed from the deformation phase signal. A multi-interferogram approach, optimally incorporating more than 30 radar scenes, is used to identify consistently coherent targets throughout the entire time series, and to derive accurate phase-change data for each target. This is facilitated through the use of “zero-baseline steering” which estimates the geometric phase contribution of different-baseline radar scenes and corrects this phase component relative to a reference or “master” scene. The identification of stable scatterers is carried out by analyzing the time series of the radar amplitude values, and by looking for persistent, bright radar reflectors, most commonly fixed dihedral structures, such as buildings or other similar objects. False phase-change signals (artifacts) due to atmospheric contributions are estimated through the use of an atmospheric phase screen (APS) analysis. Atmospheric phase contributions are determined for each radar acquisition and subtracted from the total phase residuals derived from the interferometry process. On the other hand, some limitations of the PS approach are the following [3]:

1) The displacement data represent the 1D LOS projection along the sensor-target Line Of Sight of a deformation that can actually occur in all three dimensions;
2) A limited range of displacement velocities (usually up to 10 cm/yr) are detected;
3) The versatility in terms of positioning of the measurement points and revisiting time is limited. A reliance on natural benchmarks implies that their position cannot be chosen freely in advance; it is still difficult to anticipate the PS density in rural areas without carrying out at least several processing steps on a significant number (15-20) of SAR images. Vegetation and snow cover at high altitudes result in PS low density;
4) The PS density drops to zero in the absence of rock outcrops and/or at least isolated man-made structures.

In total 614 ERS 1 & 2 scenes acquired between May 1992 and January 2001 by the ESA (European Space Agency) sensors along descending and ascending orbits were used for the interferometric analysis of the Piemonte Region. About 2 million PS were identified in the descending scenes, while only 300,000 PS were extracted from ascending scenes. This was mainly due to the limited number of the images acquired along ascending orbit and the unfavorable temporal and geometrical (satellite baseline) distribution of the acquisitions. In the Piemonte, PS typically correspond to man-made structures such as buildings, antenna and to natural reflectors, such as boulders, debris or bare rock.

The geocoding accuracy of the PS locations is around ± 6-10 m in the easting and ± 2-5 meters in the northing direction, while the estimated accuracy of the elevation values is usually better than 2 m [16].

The LOS (Line of sight direction) displacement rates (VLOS) have a precision usually better than 0.1-2 mm/yr, depending on the amount of available data, the local PS density (a key element in the estimation of spurious atmospheric phase components), and the distance from the reference point.

The standard PS analysis (SPSA) was employed for the whole territory, it allows the detection of radar benchmarks and estimation of their average velocity during the monitored period through an automatic procedure. A linear motion model is searched for and information about linear velocity is extracted. This approach is suitable for processing large numbers of scenes related to wide areas in a limited period of time. The results of this analysis consist of the yearly average velocity of a series of points relative to a reference point supposed motionless.

The time series of the LOS displacements (with respect to the reference PS) were recovered for 10 percent of PS. The precision on single measurements is related to coherence and ranges from 2 to 5 mm [16].

## 4. Methods for geological interpretation

**Figure 2.**
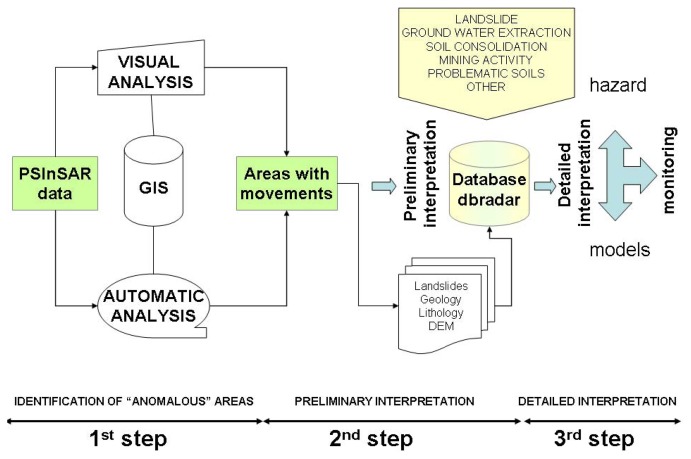
Flow chart showing the study method.

The high number of PS data (more than 2,000,000) and the large extension (25,000 km^2^) of the study area require the development of a methodology for the geological interpretation of the PSInSAR™ data at regional scale. A methodology, easy to use by the public administrations and by civil protection authorities, is here proposed (Figure 2).

**Figure 3.**
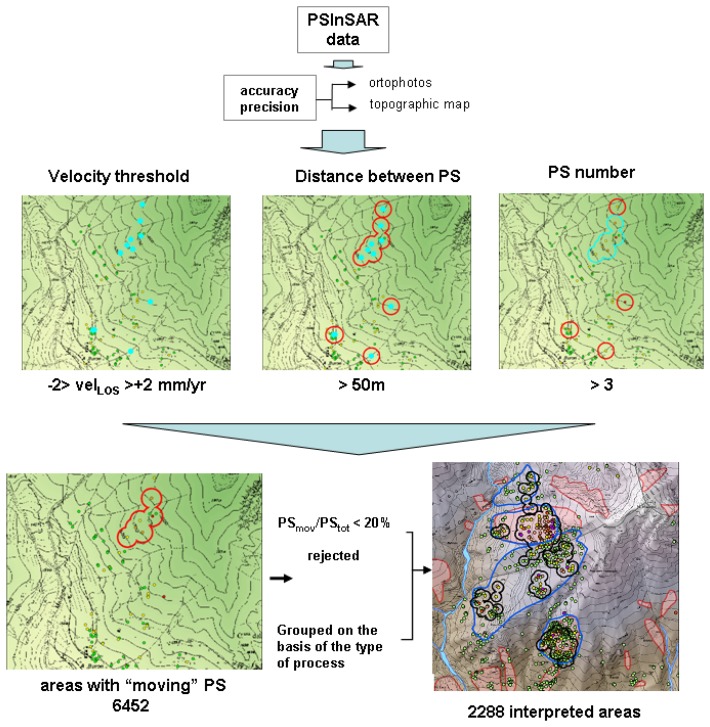
1^st^ step of the methodology used for the PS geological interpretation: identification of anomalous areas.

The PSInSAR™ data interpretation is done into three steps. The first step corresponds to the deformation accuracy assessment and the identification of the areas with significant movements, the so-called “anomalous areas”. Ortho-rectified aerial photo and cartographic data layers have been used to check the planimetric accuracy. The reference points were controlled in the field to verify if they really correspond to “geologically” motionless point. The “anomalous areas” consist of clusters of minimum 3 PS with a maximum distance of 50 meters among, characterized by displacement rates over to ± 2 mm/yr that are above a significant threshold background related to the technique precision. The identification of such areas is done through an automatic procedure in a G.I.S. environment (ArcGIS 9.1) (Figure 3). The automatic anomalous areas do not have, of course, a geological significance, but they are useful to quick and systematic identify sectors in the studied area where the PSInSAR analysis detects ground deformation and where the attention of the geologist has to be focused. More than 6,400 anomalous areas are identified in Piemonte region.

In the second step a preliminary interpretation of the anomalous areas is done through the integration in a GIS environment of the PS data with information which might have relevance in explaining the patterns of motions of PS points: 1:10,000 scale topographic maps, aerial orthophotos acquired at 1:10,000 scale during 2000, geology, Digital Elevation Models (20 × 20 m), landslide inventory and geotechnical database. Terrain slope and aspect maps were derived from the DEM for the interpretation of directions of ground movements measured by InSAR. In fact PS technology provides only the component of the real displacement vector measured along the satellite's line of sight (LOS). In order to estimate the movement direction compatible with the PS measurements in mountain and hilly areas, it is necessary to combine the LOS information (different for ascending and descending orbits) with the topographic features (e.g. slope and aspect). In addition, the sign of the measured displacements (positive values indicate movement towards the satellite along its LOS, while negative values indicate movement away from the sensor) has to be interpreted considering the terrain slope. The landslide inventory was done by ARPA Piemonte, following the Italian national methodology [17], through a detailed photo-interpretation on the whole regional area, the collection of existing data and field surveys. Classification of landslides (type, general features, *etc.*) was made referring to the international literature [18]. In order to simplify the cartographic interpretation of numerous and very small single phenomena that affected homogeneous areas an additional term was also introduced. With the term “areas affected by.” it was possible to classify slope sectors affected by seasonal widespread falls/topples and by shallow landslides/rapid flows. The evaluation of state of activity, where geological surveys and instrumental data were not available, was done through a morphological approach based on airphoto interpretation [15] of different periods; this fact complicates the comparison between the state of activity of landslides and the PSInSAR data. In the plain area the geotechnical database, containing information about the borehole and water well logs, helps the interpretation of ground deformation detected by PS.

The possible causes of movements of the anomalous areas are related to: slope instability, natural subsidence due to consolidation of soft soils or to problematic soils (e.g. swelling soils), subsidence due to rock dissolution (e.g. gypsum), subsidence due to ground water or oil-gas extraction, subsidence due to mining and underground construction, subsidence due to overimposition of some external loads (e.g. buildings), settlement of buildings due to problems related to soil foundation or/and structure, seasonal surficial movements related to talus debris, others (e.g. river bank erosion, quarry activity). The velocity threshold of ± 2 mm/yr for the identification of the anomalous areas does not allow us to identify ground movements related to neotectonic activity, which generally in Piemonte is characterized by lower displacements.

The overlying of the anomalous areas with all the other data layers allows visual grouping of anomalous areas that seem to belong to the same geological process (e.g. different anomalous areas in correspondence of the same slope instability) or to divide those areas, whose movement appears to be related to different geological processes. Finally the anomalous areas are selected on the basis of the ratio between moving PS and total PS contained in the area (generally where this ratio is less than 20 % the area is rejected). About 2,300 “interpreted areas” are obtained (Figure 3).

A GIS-based database is created containing the information about these areas: location, geological and geomorphological characteristics of the areas, SAR data (sensor, track, frame, dataset, reference point), typology of radar targets (man-made structure, rock, debris), statistical data (minimum, maximum and average coherence and LOS velocity, ratio between moving PS and total PS), and preliminary interpretation. The database is available to all potential users (professionals, public agencies, local authorities) through the ARPA Web-GIS services.

Detailed geomorphological, geological and geotechnical studies and field checks in the interpreted areas allow detailed interpretation of the displacements identified in the PSInSAR™ analysis in the third step. In this paper we present the results concerning the first and second step of the proposed methodology.

## 5. Interpretation at regional scale

**Figure 4.**
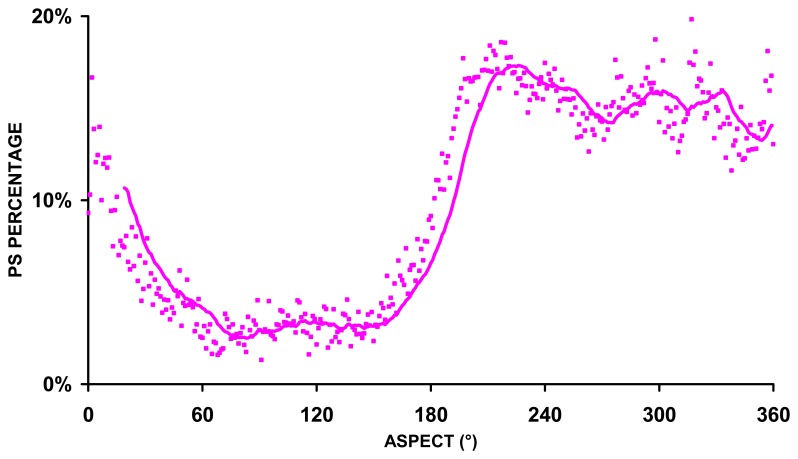
Descending PS on landslides vs. aspect.

The interpretation of the PSInSAR data depends on the typology of geological or anthopogenic processes, particularly their velocity and type of movement (vertical, horizontal, linear, non-linear). Several points must be kept in mind when interpreting the velocity values:

1. Permanent Scatterer locations and density: a feature of PS is that the number and location of permanent scatterers cannot be predicted before processing as a good ‘back-scattering’ point depends on the dielectric properties of the target materials and the planes of surfaces (geometry) in relation to the satellite. In typical built-up urban areas, one may reasonably expect many hundred points per square kilometer. However, this density falls off rapidly as more rural environments are considered (Alps and Apennines). About one-third of the regional territory has no radar coverage in relation to surface cover (presence of vegetation), high slope inclination, unsuitable orientation with respect to the SAR view angle (foreshortening and layover effects [19]) [20]. The lack of ascending data means that we cannot retrieve any information from ERS1/2 data about movements on NE facing slopes (Figure 4). The satellite orbit (approximately N-S) limits the technique's capabilities for monitoring processes with N-S direction of movements.
2. Relative movements: all values are relative to one of the arbitrarily chosen reference points that are assumed to be stable;
3. Distance of the reference point: the standard deviation of velocity errors increases with distance from the reference point. The reference points could be quite distant from the area of interest;
4. ‘Multi-path’ reflections: a proportion of PS measurements might in fact be reflections from buildings as opposed to the ground, or indeed the result of ‘multi-path’ reflections (e.g. from satellite to pavement, to building, to another building, then back to satellite). These phenomena should be taken in account, especially when considering the nature of individual, or ‘spurious’ points;
5. Velocity along the line -of-sight to the satellite: the velocity given is the velocity along the line -of-sight to the satellite, which, for ERS satellite, is on average 23° from the vertical. If the true movement direction is not along this line-of-sight, the velocity is an underestimate of the true velocity. This is especially true if there is a large horizontal component (e.g. translational slide).
6. High displacement rates: InSAR phase measurements are recorded as wrapped phase in the range -π to +π. Phase unwrapping solves this ambiguity by calculating the correct number of phase cycles that need to be added to each wrapped phase measurement so that the correct slant range distance can be computed. However in areas where more than one phase cycle of movement has occurred between sampling, i.e. between contiguous satellite acquisitions, the phase unwrapping may fail. The ambiguity of phase measurements implies the impossibility to track correctly and unambiguously a single PS LOS deformation exceeding λ/4 (=1.4 cm for ERS) within one revisiting time interval (35 days for ERS), i.e. approximately 14.5 cm/yr. In practice it is extremely difficult to detect LOS displacement rates exceeding 8-10 cm/yr. Areas of such high displacement rates (e.g. areas of active mining) can appear as regions devoid of PS points or indeed points where the displacement rate given is inaccurate [19];
7. Significantly non-linear motion: the standard PS process, used in Piemonte region, assumes ground motion that is largely linear in nature, and the standard algorithms employed may therefore average non-linear motions. Where significant non-linear motion is suspected (e.g. seasonal movements), the algorithm employed may be modified, e.g. through application of polynomial models or a reduction in the coherence threshold [21];
8. Different measurement philosophies between the PS approach and the in-situ monitoring: in-situ conventional monitoring systems are strategically located, whereas the PS position cannot be chosen freely in advance. The PSInSAR performances depend on SAR image availability (temporal sampling) and on PS availability (spatial sampling).

The main causes of movements detected by PS data in Piemonte region are represented by landslides (15% - 20% of interpreted areas respectively in the Alps-Apennines and in the Langhe sectors). Settlement of buildings due to problems related to soil foundation or/and structure represents the second cause of ground deformation (7% of interpreted areas in the Alps, 12% in the Apennines and the 20% in the Langhe and in the plain sectors). Subsidence due to overimposition of some external loads is diffused in the plain area (14% of interpreted areas) and in the Langhe (13%). 27 % of the interpreted areas in the Alps correspond to movement related to debris. Limited cases are related to subsidence due to ground water extraction (plain area), to rock dissolution (Apennines) and to consolidation of soft soils (valley bottom in the Alps). The capabilities of the technique in the landslide and subsidence detection in Piemonte region are presented and discussed In the next paragraphs.

## 6. Landslides

In order to highlight the percentage of information coming from the PSInSAR™ technique and to evaluate its effectiveness in slope movement identification a simple statistical analysis of the number of landslide with PS information was firstly performed. For this purpose the PS were overlaid upon the pre-existing IFFI landslide inventory.

**Figure 5.**
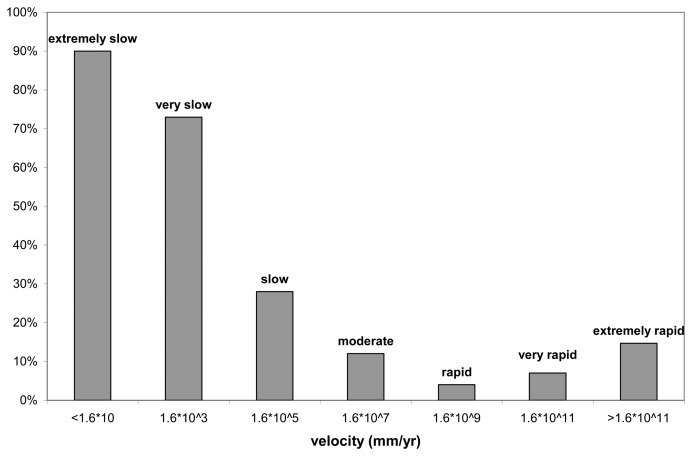
Landslides with at least 1 PS vs. landslide velocity.

The landslides with PS information are represented by extremely slow to slow movements (from a few millimeters to several centimeters per year), for which the 90% have at least 1 PS (Figure 5). In the alpine region 20% of the landslides have PS information. 12% and 8% of the originally mapped landslides have PS information respectively in the Apennine and in the Langhe areas. These are quite good results compared with similar studies, e.g. Farina *et al.* [4] in the Apennines (Arno Basin) found only 6% of the landslides with PS information.

Next, the interpreted areas were overlaid upon the landslide inventory. 30-40% of them were within or close to mapped mass movements and could also give information about the state of activity of the landslides in the period 1992-2001. Only 2-3 % of the interpreted areas correspond to possible new mass movements.

The 30% of monitored landslides had PS information and the monitoring period corresponds with that of the interferometric analysis for only a few of them.

In order to illustrate the capability of the technique to identify different mechanisms of movements related to slope instabilities some cases histories related to the main typologies of landslides in Piemonte in relation to the different geological context will presented.

### 6.1. Large landslides in the Alps

In the Alps about 20% of the landslides carried PS information, 14% of landslides with PS information had anomalous areas; they correspond to complex landslides and to deep-seated gravitational slope deformation (DSGD), that are considered as large landslides with a surface extension greater than 0.2 km^2^. The observed movements are generally from extremely slow to slow; they are fairly regular with some occasional acceleration. Secondary landslides (rock falls, toppling, rock-slides, debris flows, rock avalanches) are often associated and they result in significant direct and indirect damage. Their surface extension and gentle morphology induce the population to use this apparently favorable land for settling villages and building infrastructure such as roads. The probability of a critical global movement of the whole mass is very low, whereas the consequences may be catastrophic. The risk induced by such phenomena is especially related to serious climatic events of high intensity or long duration, which may bring about a momentary acceleration and an increase of movements. It is difficult to characterize their boundaries, their rates of movement and to understand their kinematics. Due to their extension and low rates of movement, which are close to the detection limit of traditional monitoring equipment, they are also difficult to monitor. The landslide slope faces west and north-west, which make the exploitation of the SAR descending mode ideal for interferometric purposes.

Two examples are reported, they refer to the Chiappera landslide and to the Alpe Baranca DSGD.

**Figure 6.**
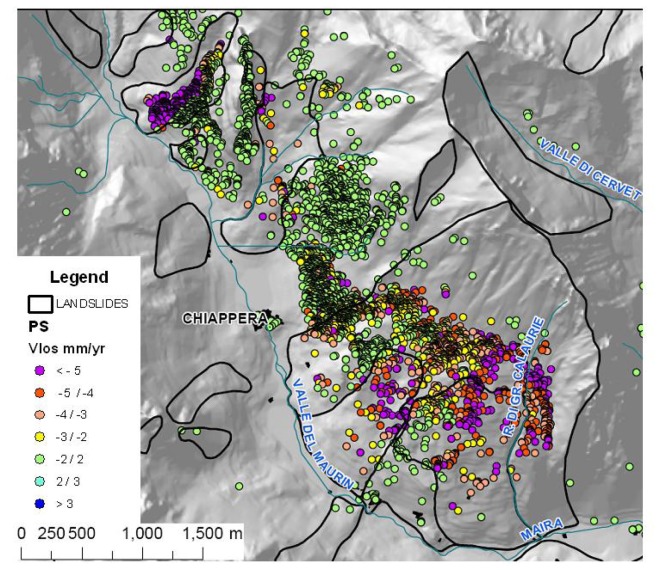
Chiappera complex landslide.

The Chiappera landslide develops between 2,000 and 1,500 m a.s.l. and it is a complex slope movement, with a surface extension of 5.1 km^2^, located on the right side of the Maira Valley (Figure 6). The slope consists on Triassic limestone, covered by talus debris and morainic deposits.

The average deformation rates along LOS (VLOS) range between − and −10 mm/yr. Thanks to the high density of natural PS targets (96 PS/km^2^) it was possible to identify some zones within the landslide characterized by different displacement rates that the geomorphological analysis had only partially highlighted.

**Figure 7.**
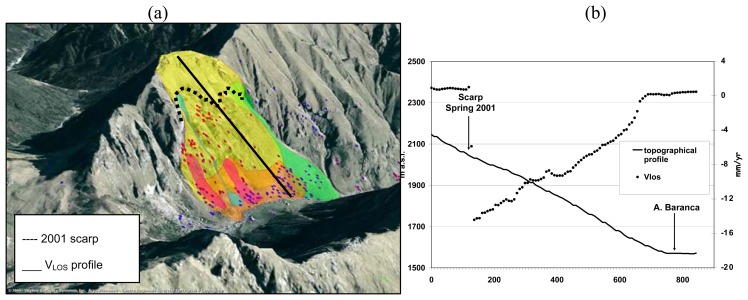
(a) Alpe Baranca DSGD. (b) VLOS profile along the longitudinal topographic profile in the Alpe Baranca DSGD.

The Alpe Baranca deep-seated gravitational deformation is located on the left side of the Mastallone Valley (Figure 7a). The geological unit belongs to Sesia-Lanzo Zone and it consists of micaschists and fine-grained gneisses, locally highly fractured. The slope shows double-crested ridges and trenches in its upper part. In the intermediate and lower part of the slope the gravitational deformation gave origin to bulging phenomenon due to rock mass dilatancy.

Figure 7b compares the change in PS LOS velocity along the longitudinal (down slope) topographic profile. The upper part of the landslide appears stable; the intermediate part moved in the period 1992-2001 at higher velocities with respect to the upper and the lower sectors. The considerable variation in PS LOS velocity in the intermediate part appears to be related to the scarp which formed in 2001. In this case PS detect pre-failure movement and have been used for planning a new monitoring system.

**Figure 8.**
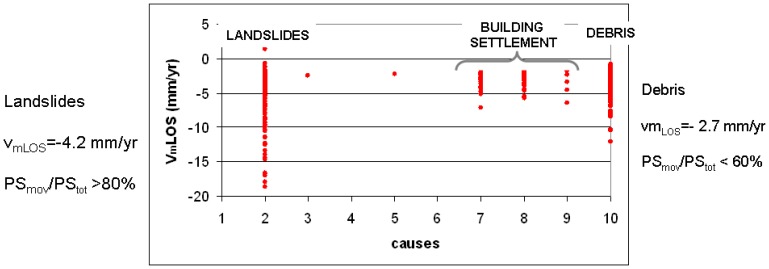
Comparison between the VLOS and the different typologies of movements in the Alps. 1: tectonic activity; 2: slope instability, 3: natural subsidence due to consolidation of soft soils or to problematic soils (e.g. swelling soils), 4: subsidence due to gypsum dissolution, 5: subsidence due to ground water or oil-gas extraction extraction, 6: subsidence due to mining and underground construction, 7: subsidence due to overimposition of some external loads (e.g. buildings), 8: settlement of buildings due to problems related to soil foundation or/and structure, 9: others (e.g. river bank erosion), 10: seasonal surficial movements related to talus debris.

One limitation of the PSInSAR technique in the Alps is that a limited number of PS corresponds to rock and the most part of PS correspond to talus debris, which is a very good reflector. This debris generally correspond to areas affected by falls/topples and explains the relatively high number of PS detected on extremely rapid landslides, as shown in Figure 5. It will be useful to distinguish between shallow seasonal movement of the debris and deformations related to deeper seated gravity. The comparison between the average VmLOS and the different typologies of movements in the Alps shows that landslides are characterized by higher velocity (up to 7 mm/yr) and higher ratio between moving PS (PSmov) and total PS (PStot) than the debris (Figure 8).

### 6.2. Rock block slides in the Langhe sector

The Langhe hills are located in the southern Piemonte. Fine-grained argillaceous rocks, including claystone, mudstone, siltstone and shale dominate the region and usually occur as alternating sequences with sandstones. The general morphology of the area is affected by the structural situation, which gives rise to long slopes dipping in the NW direction with down slope stratification as well as to short steep slopes dipping in the SE direction with counter slope stratification. The gentler hillsides are affected by “rock block slides”; the unstable mass slides along a surface coinciding with bedding planes dipping from 8° to 18° [22]. These phenomena involve the bedrock from depths of a few meters, up to 30 meters. Sliding surface corresponds to where sandy-arenaceous and marly-silty levels meet, which is the area where infiltration water is mostly concentrated. These slides took place over a period ranging from a few minutes to some hours, after strong rainfall events. Most of the observed landslides turned out to be reactivations of similar phenomena already identified in the past.

**Figure 9.**
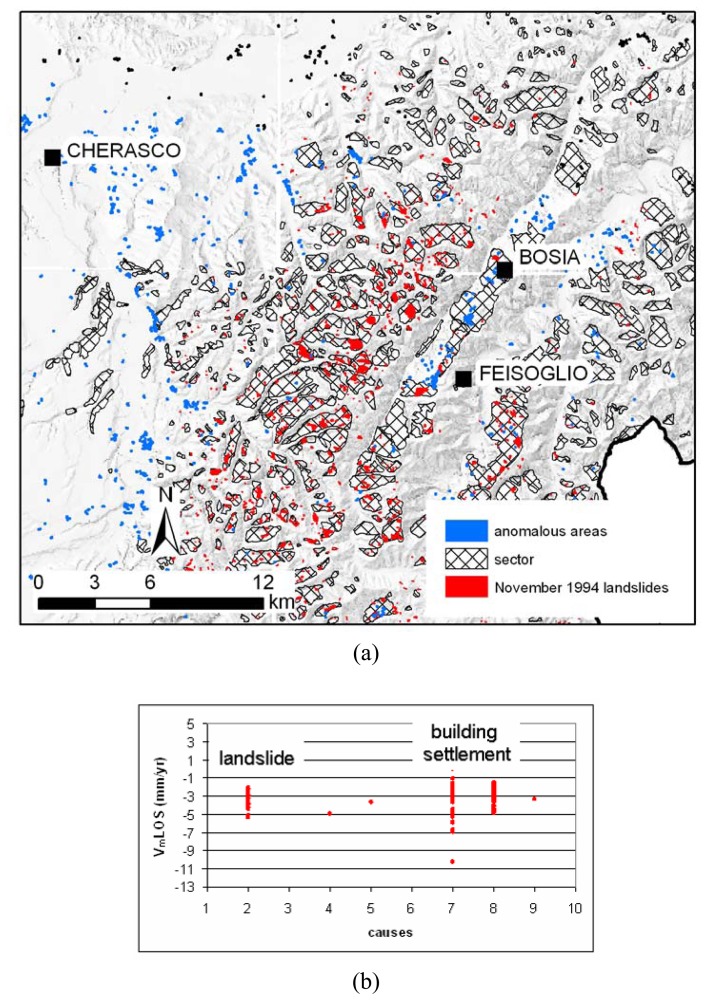
**(a)** Landslides in the Langhe area. **(b)** Comparison between the VLOS and the different typologies of movements in the Langhe (see Figure 8 for legend).

Figure 9a shows the landslides activated or re-activated in the November 1994 event [23]. Due to high deformation rates (during the peak phase, movements reached speeds varying from a few decimeters up to some hundreds meters per hour) no PS were detected on these landslides. Nevertheless some anomalous areas corresponds to the called “sectors”, which are zones with some geomorphological evidences of past landslide activities. The PS show a displacement rate of −2/–5 mm/yr, which could reflect the long term post-failure slope deformations. In the Langhe environment it could be sometimes difficult to distinguish and separate different processes, as with slope movements and the settlement of engineered structures, which have comparable velocities (Figure 9b).

**Figure 10.**
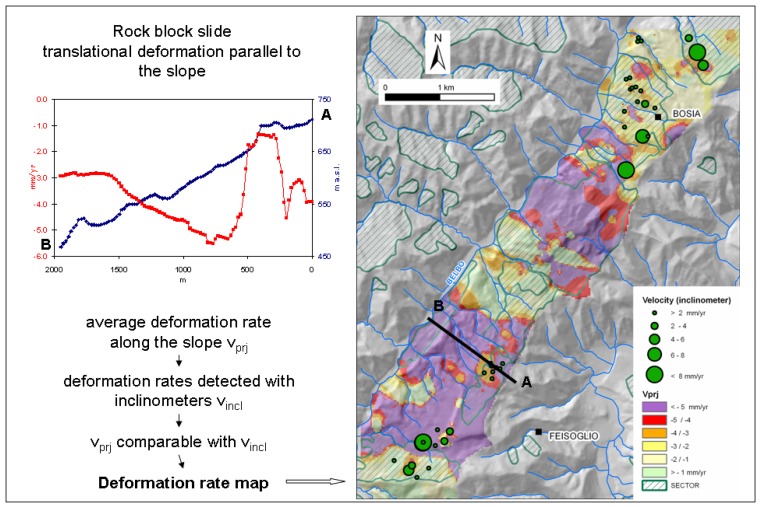
Comparison between the velocity measured by the inclinometers and the velocity of PS along the slope.

In order to survey the displacements in the Langhe region after November 1994 a monitoring system was installed, it consists in several inclinometers and piezometers. In figure 10 the velocity measured by the inclinometers in the period 1994-2000 was compared with PS measurements converted along the slope (Vprj). This was possible because the deformation (rock block slide) is translational and parallel to the slope. The average deformation rates along the slope (Vprj) range between -3 and -5 mm/yr. The PS motions are consistent with the ones inferred from in-situ measurements and a deformation rate map was obtained by interpolating the PS.

### 6.3. Complex landslides and slow flows in Apennines

The most important movements in Apennine correspond to complex landslides dominated by a rotational component in the upper part evolving to a slow flow. They generally occur on multiple shear surfaces. In general the activity of the landslides is characterized by slow continuous movements with seasonal remobilizations of slope typically related to rainfall events.

**Figure 11.**
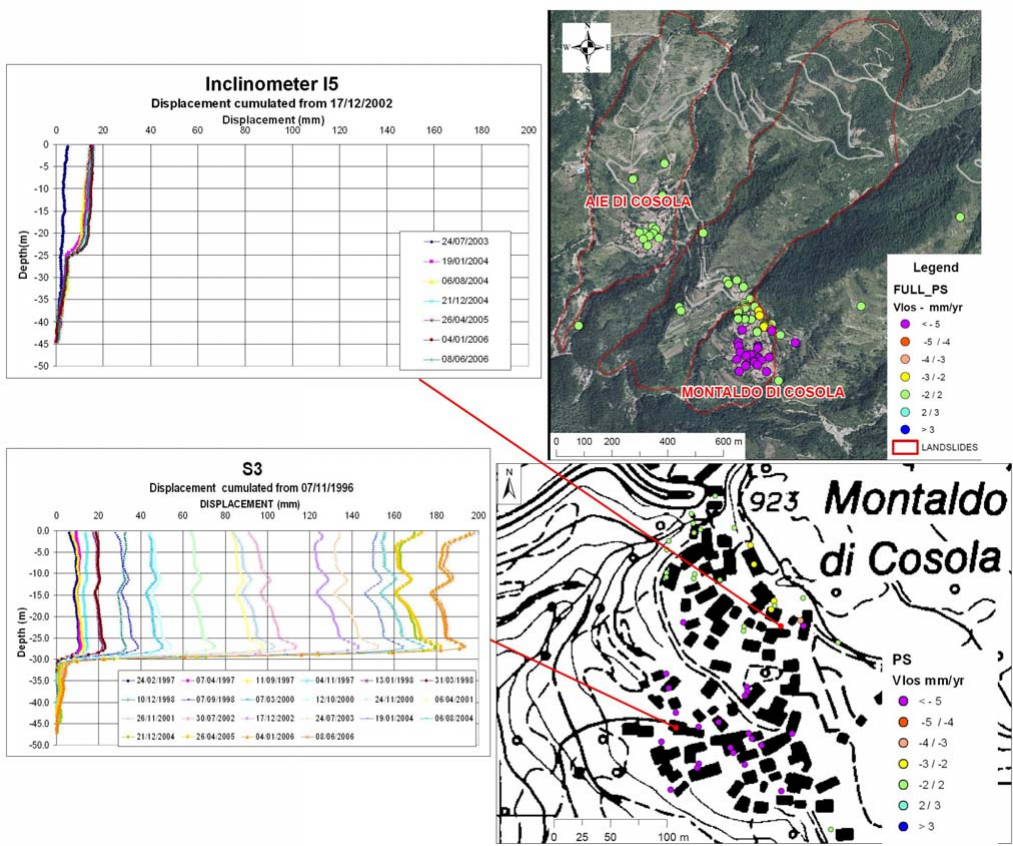
Cabella landslide.

The Cabella landslide is located in the mountain area of the Apennine and it is representative of the slow movements in this sector of the Piemonte region (Figure 11). The landslide takes place in Monte Antola Limestone and has been classified as complex. The landslide body has a thickness ranging from few meters in the upper part of the Montaldo di Cosola Village to 36 m in the southern-lower part. The most surficial sliding surface is located of about 13 m at the level of a sandy-silt layer with some clay lenses inside. The landslide has been strongly studied and the monitoring activities have been concentrated in the southern part of the Montaldo di Cosola hamlet. The monitoring network consists of five piezometers and six inclinometers, two of which provided with an Automated Inclinometer System [24]. A good correlation was found between the movements measured with inclinometers and the rainfall with a time lag of 9 days.

Due to high vegetation cover PS correspond mainly to buildings of the hamlets of Montaldo di Cosola and Aie di Cosola, then the landslide has a low PS density. PS analysis revealed that the southern part of Montaldo di Cosola is characterized by higher displacement rates than those in the northern part of the village. PS were moving at rate up to 10 mm/yr along the LOS during 1992-2001, in agreement with the inclinometer measurement and with the distribution of the damaged buildings.

**Figure 12.**
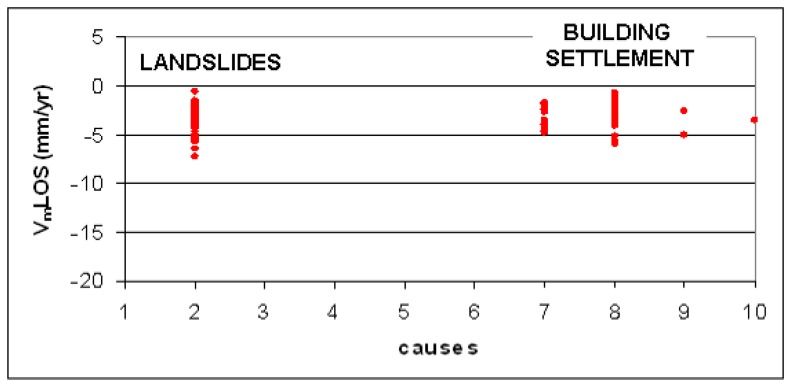
Comparison between the VLOS and the different typologies of movements in the Apennines (see Figure 8 for legend).

The study of the temporal evolution of the landslides on the basis of PS displacement is difficult in this environment because of the existence of other superimposed factors related to the subsoil processes (e.g. swelling/shrinkage of clay soils) and the behaviour of man-made structures which correspond to PS radar targets, which often present similar displacement rates (Figure 12).

## 7. Subsidence

Subsidence phenomena in Piemonte region are mainly related to two types of processes: consolidation of recent sediments and construction of buildings.

**Figure 13.**
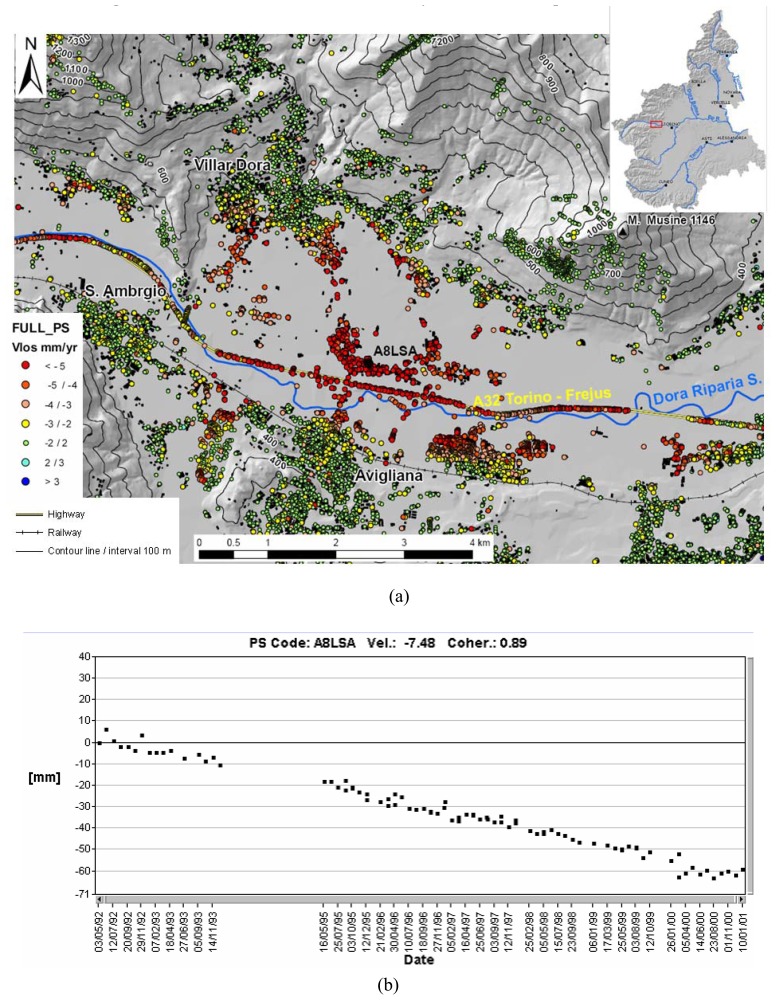
**(a)** Subsidence in the Susa Valley. **(b)** PS LOS displacement series.

Natural subsidence is related to the presence of recent sediments and an example is located in the lower part of the Susa Valley (Western Alps, Figure 13a), an area of glacial lake sediments. The glacio-lacustrine sediments have a maximum thickness of about 200 m and are characterized by gravel, sand and soft, high porous fine-grained soils. Some concentrations of organic matter and thin peat levels (representing very compressible sediments) are also present. During 1992-2001 the area was affected by subsidence in the order of −2/–5 mm/yr (Figure 13b). The PS LOS velocity increases towards the center of the valley.

**Figure 14.**
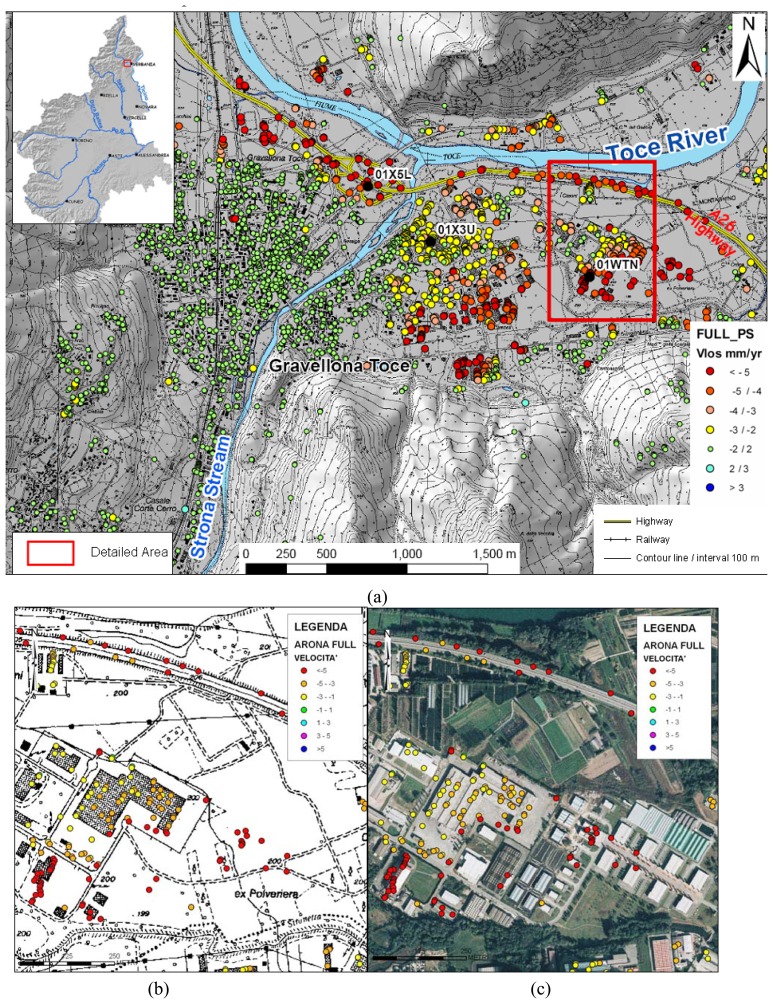
**(a)** Strona Stream alluvial fan. **(b)** Detail of the area with new buildings on the 1984 1:10,000 topographic map. **(c)** Detail of the area with new buildings on the 2000 1:10,000 aerial orthophotos.

High rate of subsidence are clustered in the terminal tract of some alpine alluvial fans. Generally the alluvial fans are strongly urbanized and therefore, they have an high PS density (> 400 PS/km^2^). An example is the Strona Stream alluvial fan near the Maggiore Lake (Figure 14a). The upper part of the alluvial fan is characterized by coarse-grained deposits (boulders of gneiss in a sandy and gravelly matrix). In the lower part and in the plain area in front of the alluvial fan the deposits are mainly finegrained soft soils (sand, sandy silt with silt); the depth to the water table is 3.5-4 m.

**Figure 15.**
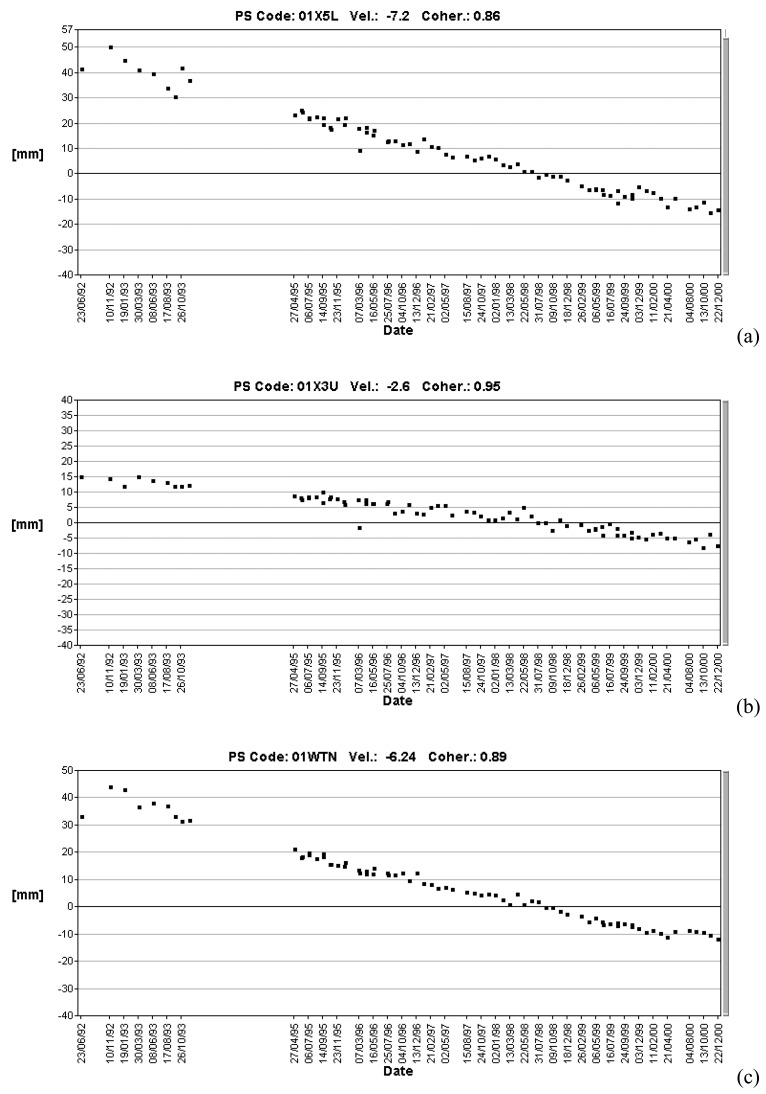
**(a)** PS LOS displacement series in correspondence of the highway. **(b)** PS LOS displacement series in correspondence of the lower sector of the Strona Stream alluvial fan. **(c)** PS LOS displacement series in correspondence of new buildings. For the location of PS LOS displacement series see Figure 14.

The PS show different LOS velocities in different areas:

1) Stable PS are located in the left side of the Strona Stream and in the upper/intermediate sectors of the alluvial fan;
2) Very high LOS velocity (< −7 mm/yr) are detected in correspondence of the highway embankment (built before 1992) (Figure 15a) and in correspondence of some new buildings (Figure 15c) in the plain in front of the alluvial fan;
3) PS with −5 mm/yr >VLOS< −3 mm/yr are present in the lower sector of the alluvial fan (Figure 15b).

The different velocities are a consequence of different lithologies in the different sectors of the alluvial fan. The stable PS (–2 mm/yr<VLOS<+2 mm/yr) correspond to the area with coarse-grained deposits. The PS with −5 mm/yr >VLOS< −3 mm/yr indicate a subsidence phenomenon related to the natural consolidation of recent soft alluvial deposits with poor geotechnical characteristics.

Faster displacements are localized at recent constructions (highway embankment and buildings). The recent alluvial deposits are the most susceptible to settle as a consequence of the overimposition of some external loads (Figures 14b and c), as the construction of buildings and roads, the imposition of man-made fills to level the ground surface, etc.

The buildings subjected to high rates of subsidence are very recent, all are built in the last 20-15 years, as it is readily inferred by the lack of a correspondence between the SAR data and the presence of buildings in the 1984 topographic map, where all these buildings are missing (Figure 14b). The PS LOS displacement series related to these buildings show a decrease with time (after 1999) (Figures 15a and 15c).

## 8. Conclusions

A method is proposed and applied to study PSInSAR results at regional scale taking into account the large amount of ERS SAR data, the extension of the study area and the great variability of geological processes.

The results show that the technique is suitable to complement and integrate information derived from conventional methods for landslides mapping in a significant, although limited, number of cases. Nevertheless, the success of the technique depends also on the typology of landslides and their related kinematics. The PSInSAR™ method is best suited for assessing the temporal evolution of slow and extremely slow landslides with constant velocity deformations (the SPSA assumes a linear model), as large landslide in the Alps. Thanks to the high PS density it was possible to identify some areas with different displacement rates. Landslides with intermittent behaviour, such as that triggered by rainfall, are difficult to detect (rock block slides in the Langhe, complex movements in Apennines), nevertheless an application of the technique could be envisaged in the detection of collapse precursors (e.g. Alpe Baranca DSGD) or post-failure movements (rock block slide in some Langhe sectors).

The PS density depends mainly on the presence of targets (rocks, debris, buildings,.), on the topographic effects (layovering, shadowing,etc.), on the vegetation and snow cover and also on the numbers of scenes elaborated, this results that in some environment, e.g. Apennine, the PS are distributed along the valley bottom where the density of man-made structures is the highest.

Another problem is the difficulty in discriminating ground deformation due to different processes, as local settlement of man-made structure (e.g. Apennine and Langhe) or the shallow deformations caused by seasonal processes in debris (Alps).

The comparison between in situ-instrumental monitoring data and the PSInSAR results requires that PS measurements are converted along the slope. This is possible for translational deformation parallel to the slope, as rock block slide. Generally there is a good agreement with the PS displacement rates and the in-situ measurements.

The PS only provides the component of the displacement vector measured along the satellite line of sight. In order to estimate the real movement it is necessary to resolve the LOS deformations with the kinematics of the slope movement (slide surface geometry). Due to the high radar viewing angles, only a fraction of the horizontal component of the movements can be detected. Therefore, a quantitative exploitation of the PS technique for the understanding of the landslide mechanism need in situ data.

In the alpine valley bottom and in the plain the technique gives very good results in the subsidence detection and monitoring, thanks to the high rate of urbanization and, therefore, the high PS density. Moderate subsidence phenomena (VLOS = 3-5 mm/yr) are related to consolidation of recent soft soils. Faster subsidence (VLOS > 7 mm/yr) corresponds to recently built areas (settlement as a consequence of the overimposition of some external loads). In this environments, being the buildings and the infrastructures the most part of detected permanent scatterers, the PSInSAR results are very usefully to detect the temporal behavior of such man-made structures.

Such regional interpretation of PS data identifies areas with ground deformations, where local authorities may concentrate risk mitigation actions, and provides the basis for additional studies at local scale (third level of the proposed methodology).

Actual and future SAR mission should reduce the current limitations. Thanks to the new (ALOS, Cosmo Sky-Med, TerraSAR-X, Radarsat-2) and planned satellite SAR missions (ESA-Sentinel 1), it will be possible to collect different data, in terms of radar response and spatial and temporal resolutions, over the same area of interest. This will allow: more frequent measurement updates; regular acquisition will allow to study more complex deformation phenomena; the increased satellite ground resolution will allow an higher PS density. Artificial reflectors (passive and actives) represents a solution for monitoring areas without natural PS.

## References

[1] Gabriel A.K., Goldstein R.M., Zebker H.A. (1989). Mapping small elevation changes over large areas: differential radar interferometry. J. Geophys. Res..

[2] Ferretti A., Prati C., Rocca F. (2001). Permanent Scatterers InSAR Interferometry. IEEE Trans. Geosci. Remote Sens..

[3] Colesanti C., Wasowski J. (2004). Satellite SAR interferometry for wide-area slope hazard detection and site-specific monitoring of slow landslides. Proc. Ninth Int. Symp. Landslides.

[4] Farina P., Colombo D., Fumagalli A., Marks F., Moretti S. (2006). Permanent Scatterers for landslide investigations: outcomes from ESA-SLAM project. Eng. Geol..

[5] Canuti P., Casagli N., Ermini L., Fanti R., Farina P. (2004). Landslide activity as a geoindicator in Italy: significance and new perspectives from remote sensing. Environ. Geol..

[6] Berardino P., Costantini M., Franceschetti G., Iodice A., Pietranera L., Rizzo V. (2003). Use of differential SAR interferometry in monitoring and modeling large slope instability at Maratea (Basilicata, Italy). Eng. Geol..

[7] Bell J.W., Amelung F., Ferretti A., Bianchi M., Novali F. (2008). Permanent scatterer InSAR reveals seasonal and long-term aquifer-system response to groundwater pumping and artificial recharge. Water Resour. Res..

[8] Chang C.P., Chang T.Y., Wang C.T., Kuo C.H., Chen K.S. (2004). Land-surface deformation corresponding to seasonal ground-water fluctuation, determining by SAR interferometry in the SW Taiwan. Math. Comput. Simul..

[9] Stramondo S., Saroli M., Tolomei C., Moro M., Doumaz F., Pesci A., Loddo F., Baldi P., Bosch E. (2007). Surface movements in Bologna (Po Plain — Italy) detected by multitemporal DInSAR. Remote Sens. Environ..

[10] Stramondo S., Bozzano F., Marra F., Wegmuller U., Cinti F.R., Moro M., Saroli M. (2008). Subsidence induced by urbanisation in the city of Rome detected by advanced InSAR. Remote Sens. Environ..

[11] Crosetto M., Crippa B., Barzaghi R. (2002). Quantitative subsidence monitoring using SAR interferometry. Proc. IGARSS.

[12] Ferretti A., Prati C., Rocca F. (2000). Nonlinear Subsidence Rate Estimation Using Permanent Scatterers in Differential SAR Interferometry. IEEE Trans. Geosci. Remote Sens..

[13] Colesanti C., Ferretti A., Prati C., Rocca F. (2003). Monitoring Landslides and Tectonic Motion with the Permanent Scatterers Technique. Eng. Geol..

[14] Ajassa R., Beretta E., Bigini E., Biancotti A., Bonansea E., Boni P., Brancucci G., Carton A., Cerutti A.V., Ferrari R., Giardino M., Laureti L., Maraga F., Marchetti G., Masino A., Motta L., Motta M., Ottone C., Pellegrini L., Rossetti R., Viola E. (1997). Mountains, hills and plains in North-Western Italy. Suppl. Geogr. Fis. Din. Quat..

[15] Colombo A., Lanteri L., Damasco M., Troisi C. (2005). Systematic GIS-based inventory as the first step for effective landslide-hazard management. Landslides.

[16] (2006). Telerilevamento Europa. Regione Piemonte report.

[17] APAT (2007). Rapporto sulle frane in Italia. Il Progetto IFFI - Metodologia, risultati e rapporti regionali.

[18] Cruden D., Varnes D.J., Turner A.K., Schuster R.L. (1996). Landslide types and processes. Landslides, investigation and mitigation.

[19] Colesanti C., Wasowski J. (2006). Investigating landslides with space-borne Synthetic Aperture Radar (SAR) interferometry. Eng. Geol..

[20] Colombo A., Mallen L., Pispico R., Ginnico C., Bianchi M., Savio G. (2006). Mappatura regionale delle aree monitorabili mediante l'uso della tecnica Ps. 10th National Conference.

[21] Colesanti C., Ferretti A., Novali F., Prati C., Rocca F. (2003). SAR monitoring of progressive and seasonal ground deformation using the Permanent Scatterers Techniques. IEEE Trans. Geosci. Remote Sens..

[22] Forlati F., Campus S., Luino F. Scivolamenti planari nelle Langhe Piemontesi: individuazione, elaborazione ed analisi di alcuni elementi significativi.

[23] Forlati F., Mortara G., Ramasco M., Susella G. (1995). Carta de-gli scivolamenti planari nell'area delle Langhe a seguito dell'evento alluvionale del 1994.

[24] Lollino G., Arattano M., Allasia P., Giordan D. (2006). Time response of a landslide to metorological events. Nat. Hazards Earth Syst. Sci..

